# DAYTIME ACTIVITY ATTENUATES ASSOCIATIONS BETWEEN SLEEP EFFICIENCY AND MORTALITY AMONG OLDER ADULTS

**DOI:** 10.1093/geroni/igae098.4151

**Published:** 2024-12-31

**Authors:** Collin Sakal, Xinyue Li

**Affiliations:** City University of Hong Kong, Hong Kong, China (People’s Republic); City University of Hong Kong, Hong Kong, China (People’s Republic)

## Abstract

Adequate sleep is crucial for promoting longevity, and yet epidemiological understanding of the relationship between sleep and mortality is largely based on a single metric: sleep duration. This study examined associations between sleep efficiency and mortality in a cohort of 33,428 older adults in the UK Biobank over approximately 9-years of follow-up. Sleep efficiency was derived from wrist-worn accelerometer data using a Hidden Markov Model-based sleep-wake detection algorithm and was summarized based on sufficiency (average) and irregularity (standard deviation) across the wear periods. Associations were examined using Cox Proportional Hazard models adjusting for sociodemographic factors, smoking, drinking, depression, social support, medications, cancer, and sleep duration. In fully adjusted models, average accelerometer-assessed total daytime activity was further included using restricted cubic splines to examine its potential to attenuate observed associations. In adjusted Cox models without daytime activity, low average (< 0.85) sleep efficiency was associated with greater risk of mortality (Ref: ≥0.85, HR: 1.31, 95% CI: 1.18, 1.46) as was high irregularity (top quartile) in sleep efficiency (Ref: bottom quartile, HR: 1.35, 95% CI: 1.20, 1.53). However, neither measure of sleep efficiency was significant after adjusting for daytime activity. L-shaped relationships were observed between daytime activity and mortality where low activity levels were associated with an increased risk and high levels of activity were associated with a decreased risk. Our findings suggest that daytime activity attenuates associations between both sufficiency and irregularity in sleep efficiency with mortality among older adults in the United Kingdom.

